# Bibliometric and scientometric analysis on biomarkers and molecular mechanisms for physical frailty and sarcopenia

**DOI:** 10.3389/fmed.2024.1326764

**Published:** 2024-02-05

**Authors:** Valentina Ginevičienė, Erinija Pranckevičienė, Justina Kilaitė, Asta Mastavičiūtė, Rūta Dadelienė, Ieva Eglė Jamontaitė, Austėja Letukienė, Ildus I. Ahmetov, Vidmantas Alekna

**Affiliations:** ^1^Faculty of Medicine, Vilnius University, Vilnius, Lithuania; ^2^Faculty of Informatics, Vytautas Magnus University, Kaunas, Lithuania; ^3^Clinic of Internal Diseases and Family Medicine, Institute of Clinical Medicine, Faculty of Medicine, Vilnius University, Vilnius, Lithuania; ^4^Research Institute for Sport and Exercise Sciences, Liverpool John Moores University, Liverpool, United Kingdom

**Keywords:** sarcopenia, frailty, biomarkers, bibliometric analysis, molecular mechanisms

## Abstract

**Introduction:**

The influence of physical frailty and sarcopenia (PFS) on the well-being of older people and continuous pressure on the healthcare systems has prompted a research on the pathophysiology and molecular mechanisms of these conditions. Nonetheless some biomarkers have been suggested as potential markers for PFS none of them have been shown to highlight the complex nature of PFS, which reveals that there is a need for an understanding of the possible biomarker candidates. The aim of this study was to identify the current research hotspots, status, and trends in the field of biomarkers and molecular mechanisms for PFS.

**Methods:**

The bibliometric and scientometric analyses were performed using VOSviewer (version 1.6.18) and open source software platform Cytoscape v.3.9 (for visualizing and constructing a network of keywords). Data of publications (from 1997 to 2023) related to biomarkers and molecular mechanisms of PFS were obtained (in May 2023) from the database of Science Citation Index Expanded of Web of Science, Scopus, and PubMed. The keywords obtained from the Scopus database were used to perform a meaningful keyword analysis. A network of keyword relationships was build using Cytoscape.

**Results:**

In this study, we present biomarker keywords for PFS in relation to other keywords potentially designating processes and mechanisms and reveal the biomarker identities and current contexts in which these biomarker identities are discussed.

**Conclusions:**

Over recent years, scientific interest in the field of PFS has increased and focused on the inflammatory process and probably will be concentrated on myokines (such as cytokines and small proteins) that are synthetized and released by skeletal muscles in response to physical activity. Moreover, proteomic and genetic markers are deeply involved in PFS.

## 1 Introduction

Physical frailty and sarcopenia (PFS) are two increasing age-related health problems worldwide, which are viewed as a crucial risk indicators of adverse health related outcomes, such as gait disturbance, falls, the need for long-term healthcare, and death (1, 2). The European Working Group on Sarcopenia in Older People revised the definition of sarcopenia in 2019 (3). According to the current definition, sarcopenia is a muscle illness defined by weak muscle strength, low muscle mass, and poor physical movement. According to Frailty Consensus from 2013, frailty is defined as “a clinical state in which there is an increase in an individual’s vulnerability for developing an increased dependency and/or mortality when exposed to a stressor” (4). Frailty can be viewed as a broad term that includes social, physical, and psychological frailty. Physical frailty “is described by reduced endurance, muscle strength, and physiological function that increases an individual’s vulnerability for developing increased dependency and/or death,” and Fried’s criteria serves as a diagnostic tool for this medical syndrome (5). In this paper, when the term frailty is used it means physical frailty.

These two conditions share a common clinical picture (such as muscular atrophy) and a number of common risk factors (such as gender, age, physical inactivity, nutritional inadequacy) (1–3, 6–8). A loss of skeletal muscle is a key component of PFS, and can lead to decreased mobility and a higher risk of disability among older adults. The association between frailty and sarcopenia and the root causes between them were still not fully grasped. Recent studies have suggested that sarcopenia could be a factor in modifying frailty status in older adults, e.g., regressing from frailty to prefrailty or from prefrailty to robustness (9, 10).

In the recent times, a higher number of research studies concentrating on the regulatory mechanisms of aging and the pathogeneses of PFS have been published. Moreover, the search for biomarkers of PFS enabling early recognition and monitoring of the progression or regression of these conditions over time is ongoing (11). Fastly growing number of publications may overwhelm the researchers interested in the field of biomarkers and their relationship with both frailty and sarcopenia, and prevent them to investigate the complex health matters in this field. To recognize the growing interest in these geriatric conditions and perform further research in the quest of biomarkers (and potential signaling pathways) for PFS, it is necessary to intensely analyze the studies in this scientific field, and bibliometric (scientometric) analysis is an appropriate way to fulfill these criteria. Bibliometric analysis (based on relevant information about scientific data and publications) can capture the state of ongoing research situation and qualitatively and quantitatively predict trend in future research fields (12, 13). Currently, bibliometric analysis has been vastly applied in key analysis of different illnesses, providing a standard for future research topics on disease treatment and prevention (12, 14, 15). In this setting bibliometric analysis would be helpful in understanding were the future of research on PFS is going. As PFS affects more and more older adults around the globe it is very important to know what pathophysiological aspects are focused on as probable prevention and treatment strategies might be implemented in near future.

As far as we know, there are still no research and articles with bibliometric analysis on the biomarkers for PFS, and only a few bibliometric studies in certain aspects of sarcopenia (such as sarcopenia and nutrition, sarcopenia and surgery, physical activity and sarcopenia) have been published (16–20). Therefore, the aim of this study was to identify the current study hotspots, status, and trends in the area of biomarkers and molecular mechanisms for PFS (based on bibliometric analysis and visualization technology).

## 2 Materials and methods

This study was performed in accordance with the principles of the Declaration of Helsinki. The research model is a situation (biomarkers and molecular mechanisms for frailty and sarcopenia) analysis study, which is one of the qualitative and/or quantitative research methods. Visualization of similarities (VOS) viewer (VOSviewer version 1.6.18) was used for performing bibliometric (scientometric) analysis.

### 2.1 Bibliometric analysis using VOSviewer

Bibliometric research involves the network analysis of articles, journals, authors, or keywords. Such co-word networks are studied by using mapping and clustering techniques (21). VOSviewer is a software tool for building and visualizing bibliometric networks. Technically, the VOSviewer creates a map of the keywords based on their similarity metrics that is constructed from the co-occurrence matrix of the most relevant words extracted from the subset of the articles (21). A strength of the connections between the keywords as occurring together in articles is reflected by the distance between the keywords on the 2D map in the Euclidean sense (proximity in x and y coordinates). The mapping objective is to place the items so that the distance between them reflects their similarity as much as possible. In addition, the VOSviewer performs a modularity-based clustering (22). This technique is based on the simultaneous use of the similarity information between items to perform mapping and assign items meaningfully to the clusters of the closest interconnected items. As a result, one obtains a semantically meaningful clustering of the keywords. Such clustering allows to see the distinctive themes characterized by the keywords in the literature on the particular topics which in our case were biomarkers and molecular mechanisms of PFS.

### 2.2 Search strategies, eligible criteria, data sources, and collection

Data of publications (from 1997 to 2023) related to biomarkers and molecular mechanisms of PFS (published in English) were obtained (in May 2023) from the database of Science Citation Index Expanded of Web of Science (WoS), Scopus, and PubMed. The following search strategies were presented: TITLE-ABS-KEY = [(physical frailty OR sarcopenia) AND (biomarkers) AND (molecular mechanism)] was used to explore and select appropriate articles (published over the past 25 years). We filtered out the search to only research articles (reviews, original articles and proceedings papers) and excluded other types of academic papers and duplicate articles from our analysis. Sorted publications and data were stratified and systematically assessed using Microsoft Office Excel 2016 (Microsoft Corporation, Redmond, WA, USA). The number of studies was evaluated by research area, authors, organizations, country, journal, and publication year. After that the percentage of the total number of articles for each category was calculated. The files containing data on the topic were imported into the VOSviewer software to conduct bibliometric analysis and data visualization. According to the setting parameters by VOSviewer a step by step drawing overlay network visualization map was created.

We generated co-word network maps of keywords, research authors, and organizations. A strength of the connections between the keywords as occurring together in articles is reflected by the distance between the keywords on the 2D map in the Euclidean sense (proximity in x and y coordinates). The frequency of publications or keywords was visualized in the map by the size of the circle nodes, associations (such as co-occurrence) were expressed as the link between nodes, and the degree of association was shown by the distance between nodes. Different clusters were shown as different color nodes in the cluster analysis and the number of clusters varied depending on the similarity threshold between nodes. The sum of the link strengths of one node over all the other node was calculated and expressed as total link strength of a node. In this study clusters were grouped automatically and the clustering resolution was appropriately adjusted if required. We used an open source software platform Cytoscape v.3.9 for visualizing complex networks and constructing a network of keyword relationships (23). We intended to determine the co-occurrence of keywords and collaboration of authors between different clusters. In addition, we noted the research trends by logging them based on the average publication year and the number of citations. This analysis helped to search for relevant research topics in the field of biomarkers and molecular mechanisms in PFS.

## 3 Results

### 3.1 Analysis of publications from the Web of Science database

**Figure 1:**
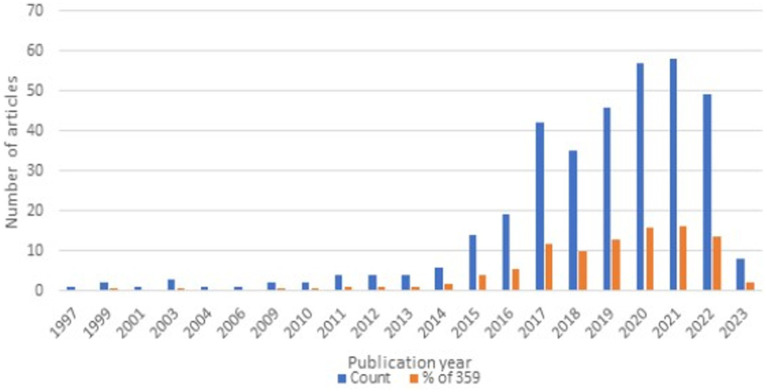
Publication trends in “physical frailty and sarcopenia” topic (WoS accessed May 2023).

Using VOSviewer we analyzed the main areas of interest in scientific research related to biomarkers and molecular mechanisms of PFS. Search on the WoS by keywords “physical frailty and sarcopenia” returned 359 results published from 1997 (first publication) until May 2023. The significant steady increase in published articles on the topic of PFS starts in the year 2015 (as shown in Figure 1).

The most cited top 20 publications have a minimum of 152 and up to a maximum of 270 citations. High citation of quite recent papers published in 2017–2019 shows very active research interest in the PFS topic. The most active research areas on the terms of “physical frailty and sarcopenia” reported by WoS relate to Geriatrics and Gerontology; Nutrition and Dietetics; General Internal Medicine; Gastroenterology; Hepatology; Endocrinology; Metabolism; Cell Biology; Biochemistry; Molecular Biology; Neurosciences; Neurology; Surgery; Cardiovascular System; Cardiology. The most cited topic by WoS in this area was “Nutrition and Dietetics” and the second most cited – “Musculoskeletal Disorders.”

Among the 354 scientific journals, the contribution of the list of top-cited articles was made by 25 different WoS indexed journals. The most articles were contributed by the “Aging Clinical and Experimental Research” journal (*n* = 21), after it came “Experimental Gerontology” (*n* = 16). “Nutrients” and “Journal of Nutrition Health Aging” each provided 13 and 11 articles, respectively. Among the 25 journals, 14 journals submitted more than or equal to 5 articles, 10 journals provided 4 articles, and 1 journal provided only 3 articles.

The country of origin and the institute of research were determined according to the first corresponding author. Countries most actively publishing were the United States of America (*n* = 82, 23.2%), followed by Italy (*n* = 80, 22.7%), and Japan (*n* = 45, 12.7%). Five corresponding authors (from Italy) published more than 30 articles each: Marzetti E. and Calvani E. published the most articles (48 and 41 articles, respectively), followed by Landi F. (40 articles), Bernabei R. (34 articles) and Picca A. (30 articles). The above-mentioned authors collaborate in SPRINTT (Sarcopenia&Physical FRailty in older people: multi-componenT Treatment strategies) Consortium.

Since our main interest lies in the molecular mechanisms and biomarkers of PFS we filtered further the 359 articles by “biomarkers.” That reduced number of articles to 44. The query “physical frailty and sarcopenia and biomarkers” filtered by “molecular mechanism” resulted in four publications indexed in WoS (24–27).

### 3.2 Analysis of publications from the SCOPUS database

**Figure 2:**
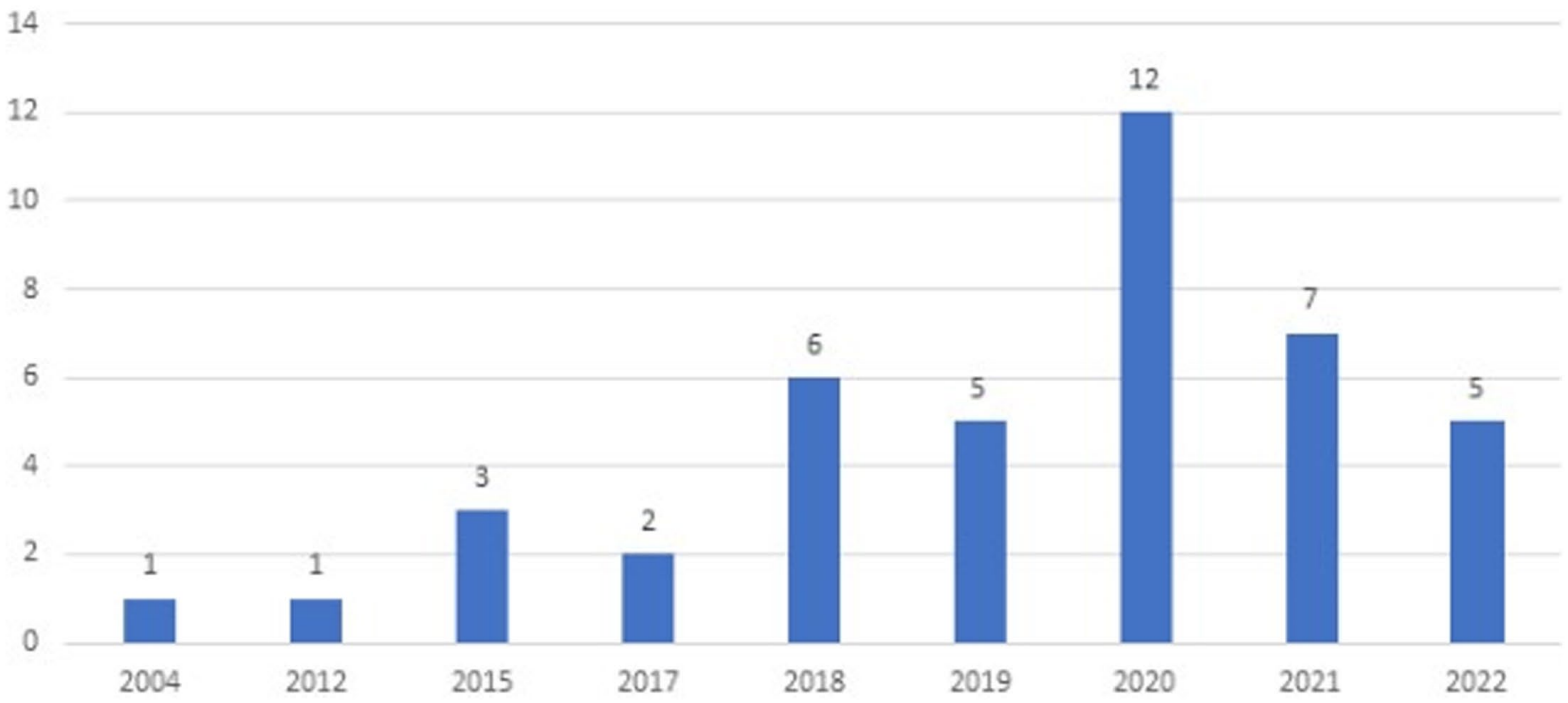
Number of publications about sarcopenia, frailty, and biomarkers in depicted years (Scopus accessed May 2023).

The same analysis was conducted in the Scopus database querying keywords, title and abstract in scientific articles published over the past 25 years (1997–2023). A query “physical frailty and sarcopenia” resulted in 250 articles in big part overlapping articles obtained in WoS database. Further filtering these 250 publications by the “biomarker” keyword returned 70 articles and filtering further by “molecular mechanism” reduced the number to 38 articles. The most cited articles in Scopus are the same articles that are most cited in WoS. There were no significant differences in the major research topics or authors. In general, the number of publications has steadily increased through the years and reached a peak in 2020 as shown in Figure 2.

Thus, the query used in Scopus resulted in more articles (*n* = 38) than the same query in WoS database (*n* = 4). Scopus database is oriented toward multidisciplinary (technical) literature and the content of the same article in Scopus may be indexed in a different manner than in the WoS database and for this reason, the query of interest resulted in more articles that it resulted in WoS.

### 3.3 Analysis of publications from the PubMed database

**Figure 3:**
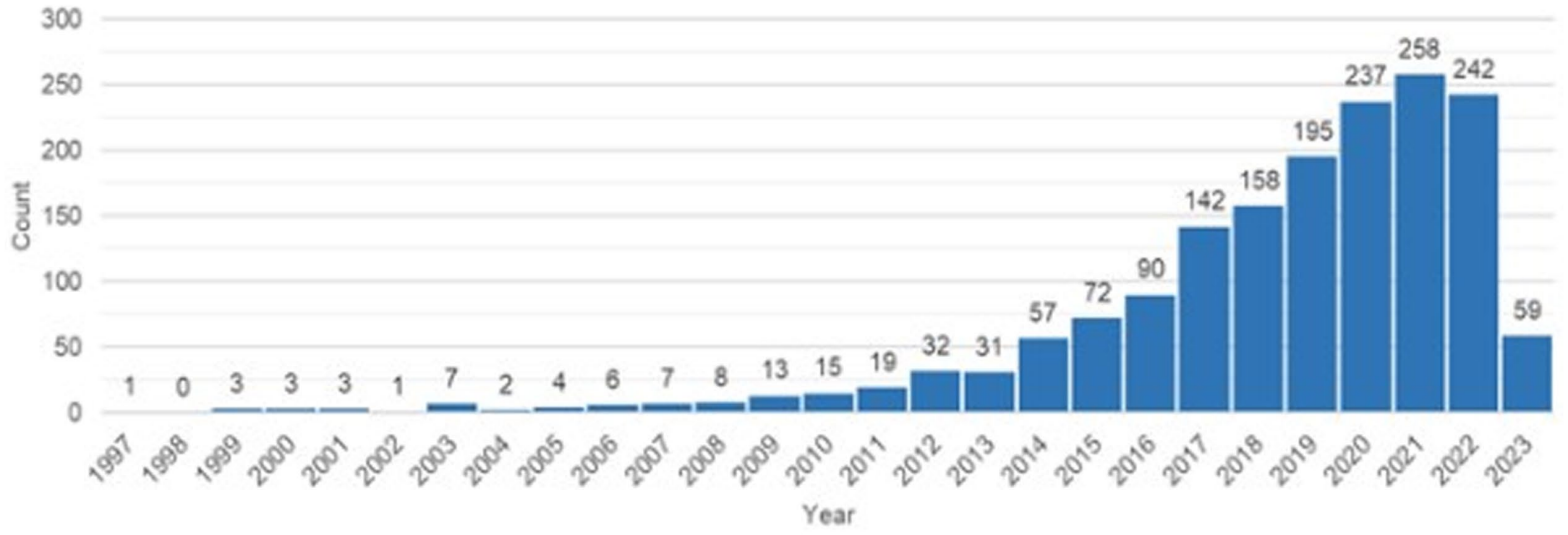
Publication trends in “sarcopenia and physical frailty” topic (PubMed accessed May 2023).

Additionally, this study attempted to expand previous bibliometric analysis (in WoS and Scopus) by examining the research status of molecular mechanisms and biomarkers for PFS in academic literature in the PubMed database. A query keywords “physical frailty AND sarcopenia” resulted in a total of 1,480 articles (which included original research, review articles, case reports, and clinical studies or clinical trials) and were published from 1997 (first publication) to 2023 (Figure 3).

Further filtering these 1,480 publications by 3 keywords (“physical frailty” OR “sarcopenia” AND “biomarkers”) returned 150 articles. The first article covering all 3 key topics was published in the PubMed database in 2003. The growth of articles has been steady, reaching the highest level in 2017. The most published authors are Marzetti E. and Calvani E. (each of them published 21 publications) and Picca A. (19 articles) from Italy (Fondazione Policlinico Universitario Agostino Gemelli IRCCS). In a detailed analysis, filtering 150 articles based on 4 keywords (“physical frailty” AND “sarcopenia” AND “biomarkers” AND “molecular mechanism”) yielded 35 published articles that were identical according to the Scopus database.

In general, data in PubMed was more difficult to obtain and analyze compared with that in the WoS and Scopus databases. However, all identified data were similar as in the other two databases. Only 4 articles related to biomarkers and molecular mechanisms of PFS were found in WoS while the Scopus database retrieved 38 articles, and PubMed – 35 articles from the same query. Wherefore, the keywords obtained from the Scopus database were used to perform a meaningful keyword analysis. The keyword analysis aimed at identifying of strong relationships between keywords of biomarkers and the contexts to which they are linked (see “*VOSviewer Mapping Result Clusters and Keywords Analysis”* section).

### 3.4 VOSviewer mapping result, clusters and keywords analysis

We used VOSviewer to analyze relationships between the keywords of biomarkers and the rest in clusters computed by VOSviewer. The most frequent keywords that were not informative and similar (overlapped with our search keywords and many co-occurred among the keywords) in biomarker context for sarcopenia and physical frailty were excluded from the interpretation of relationships between keywords.

**Figure 4:**
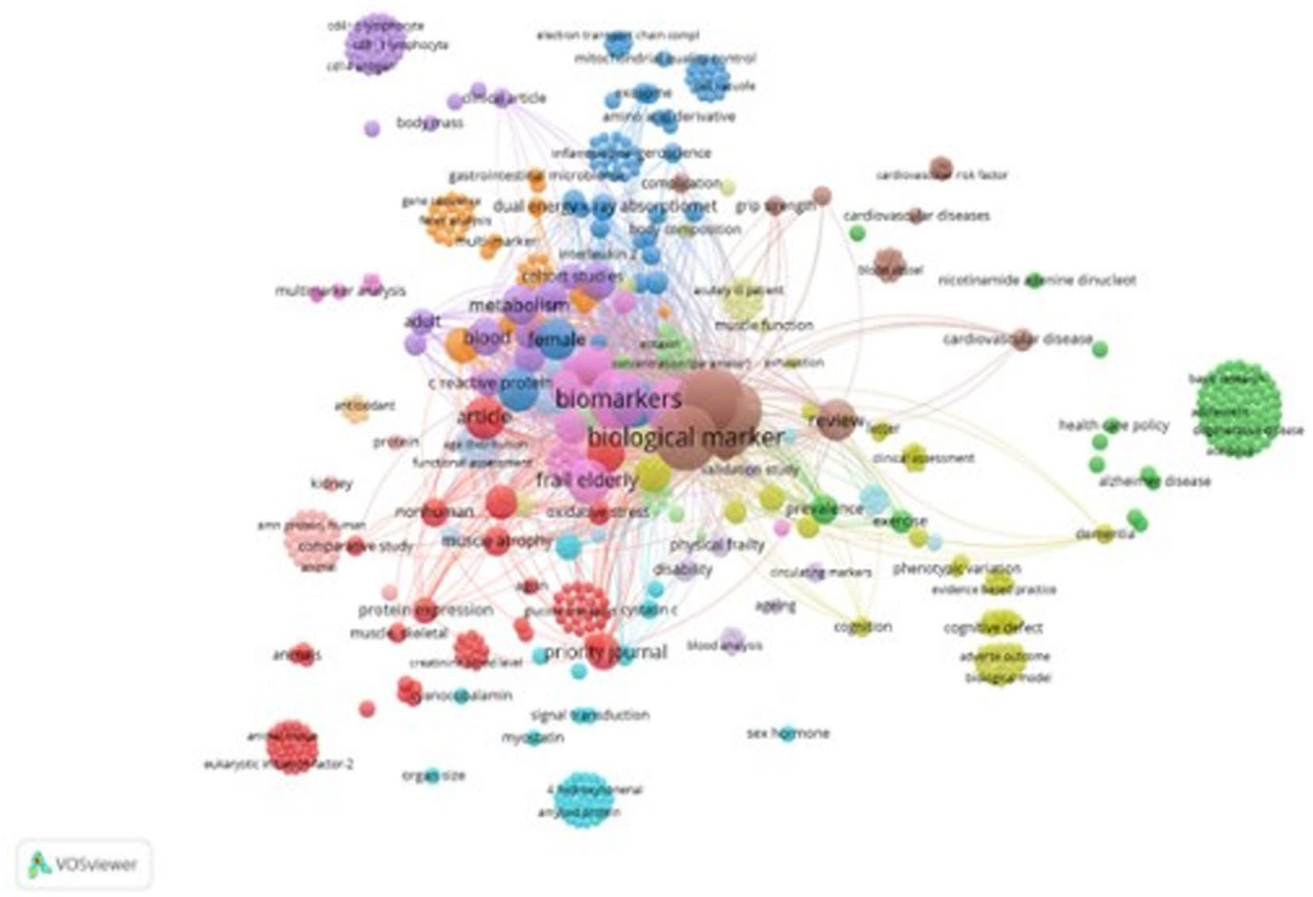
Co-occurrence network of high-frequency keywords. In the map, the size of nodes manifests the frequency of the keyword’s occurrence, while lines show relationships among keywords. The shorter the distance between two nodes, the larger the number of co-occurrence of the two keywords. Source: own study based on data retrieved from Scopus database as of 22 May 2023 and analyzed with the use of VOSviewer.

We looked specifically only into which biomarkers and in which contexts of PFS are most discussed in the literature using keywords of the Scopus document set because it returned more articles. In total 640 keywords were added to the keyword co-occurrence analysis by VOSviewer which created 15 semantic clusters depicted in Figure 4.

The 15 semantic groups of the most-occurring keywords can be generalized into some meaningful themes that are listed in Table 1.

**Table 1:** General themes revealed by VOSviewer keyword clustering based on keyword co-occurrences.

Cluster	Number of keywords	Theme
1	86	Muscle function, signaling pathways, myokines, proteomics, biomarkers
2	78	Health care, infectious diseases, immunology
3	71	Inflammation, aging, biomarkers
4	61	Cognition
5	51	Physical performance, immunology
6	43	Muscle growth, tissues, biomarkers
7	40	Nutrition, metabolism, intestinal flora
8	36	Genetics, SNPs (single nucleotide polymorphisms), grip strength
9	35	Community living, cellular senescence, SASP (Senescence-associated secretory phenotype)
10	30	Metabolism, metabolites, nutrition
11	25	Inflammaging, immunosenescence, very elderly
12	24	Cardiovascular health, functional assessment
13	23	Geriatric medicine, muscle function
14	17	Frailty, sarcopenia, elderly care, prognosis
15	13	Frailty, falling, glomeruli, glomeruli filtration rate

The most-occurring keywords expressions are: “muscle function, myokines, proteomics, biomarkers” (86, cluster 1), “health care, infectious diseases, immunology” (78, cluster 2), “Inflammation, aging, biomarkers” (71, cluster 3), “Cognition” (61, cluster 4), “Physical performance, immunology” (51, cluster 5). Interestingly, the several keywords in cluster 8 were associated with various genetic terms, such as “gene,” “gene expression,” “gene sequence,” “genetic polymorphism,” SNPs, “genome-wide association study” etc. and associated with the phenotype of PFS and appears only in a few latest articles in the recent years (2017–2023).

### 3.5 Keywords network analysis

The keyword occurrences and co-occurrences help to understand contexts in which biomarkers and molecular mechanisms of PFS are discussed in articles. We applied network analysis to explore biomarker keywords and how they relate to other keywords. Only those keywords were included in the subsequent analysis that occurred together in articles at least two times.

**Figure 5:**
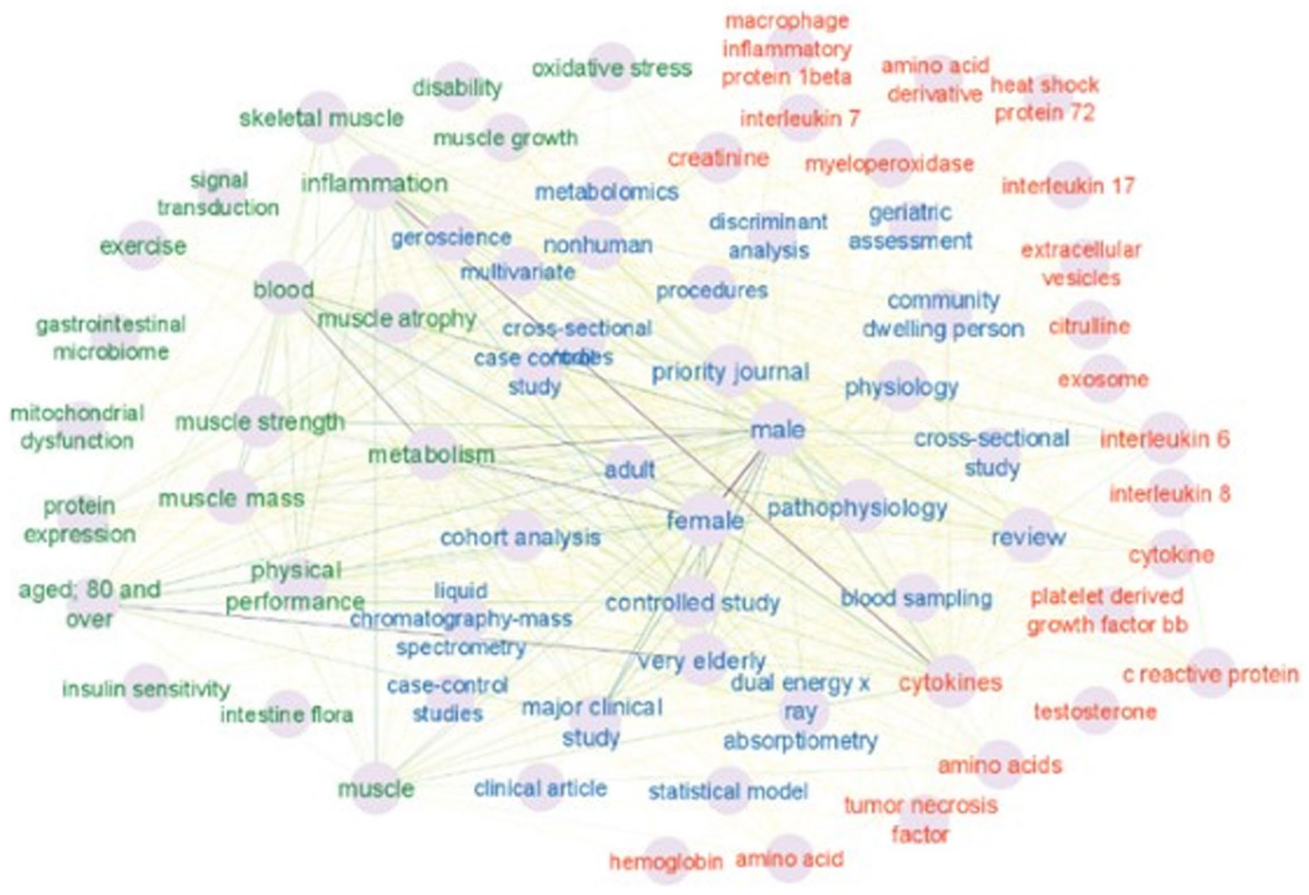
Three clusters are shown on the map. The red cluster 1 of keywords indicates bioentity. The green cluster 2 represents the physiological process. The blue cluster 3 involved resources. The intensity of the connecting lines represents a number of keyword occurrences together. Very pale connecting lines indicate that the keywords co-occurred only two times. Source: own study based on data retrieved from Scopus database.

We used Cytoscape v.3.9 to construct a network of keyword relationships. The keywords in the network were labeled manually as a bioentity, a process, or just a keyword. The bioentity label was given to keywords that represent biological molecules and entities such as “creatinine,” “amino acids,” “cytokines,” “citrulline,” “interleukin” and similar (cluster 1). The physiological process label was given to the keywords such as “inflammation,” “metabolism,” “disability,” “oxidative stress” and other similar keywords (cluster 2) that represent functions and processes that may be affected by bioentities. Other keywords like “cohort analysis,” “metabolomics,” “*dual*-*energy X*-*ray* absorptiometry,” “male,” “female,” “priority journal” were labeled as resource keywords (indicates the research methods, strategy, subjects, etc.) (cluster 3). All keywords are connected to each other. Figure 5 represents a network of all labeled keywords.

**Figure 6:**
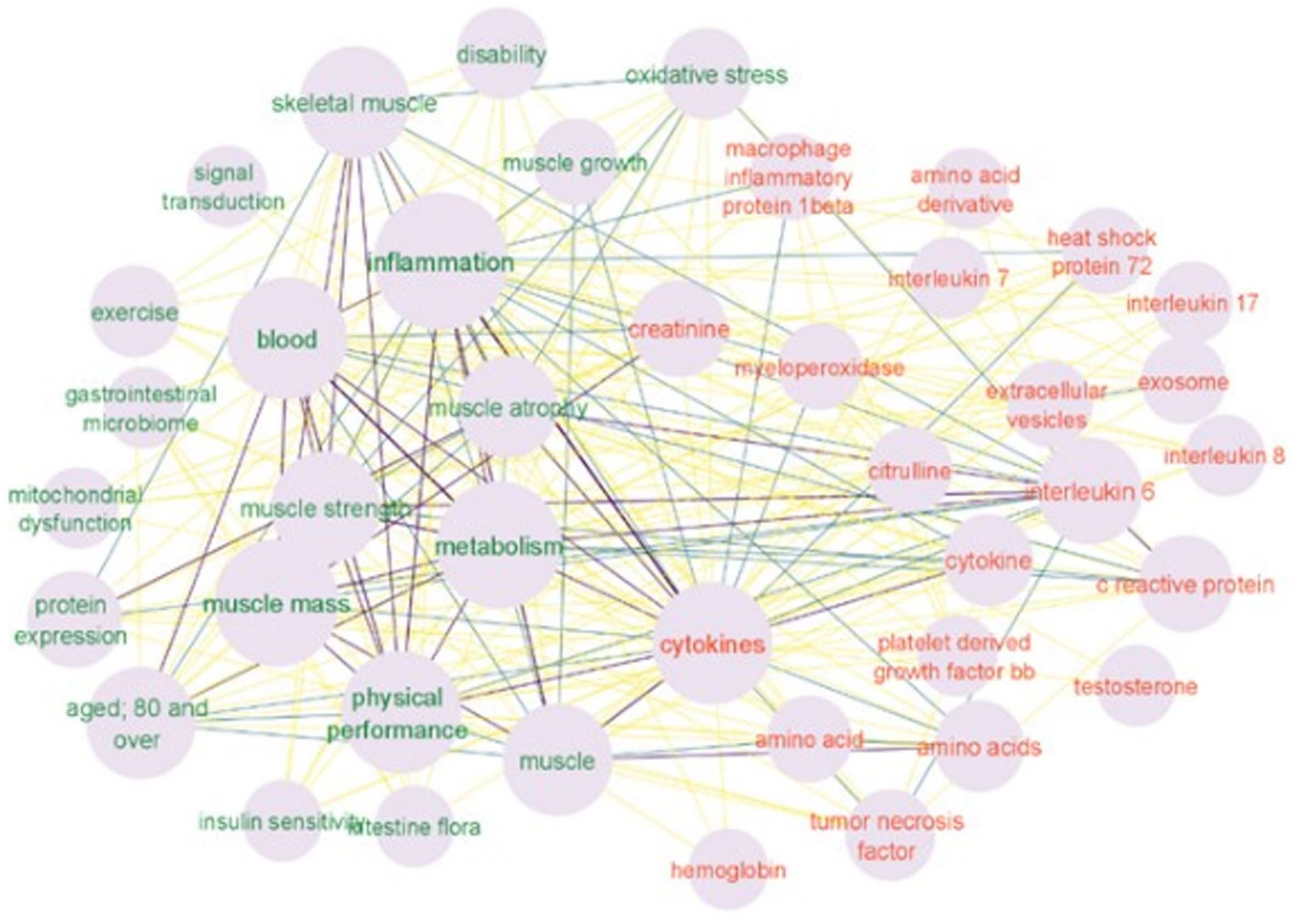
Two clusters are shown in the map (keyword network of the molecular mechanisms and biomarkers in PFS): the red cluster indicates biomarkers and the green cluster – physiological processes. The size of the node is proportional to the number of keyword occurrences. The intensity of the connected lines represents the extent of keyword pairs occurring together. Source: own study based on data retrieved from Scopus database.

Keyword co-occurrence analysis provides information about which bioentities-biomarkers are discussed in the literature, and which processes and other biomarkers they connect to. In order to extract these relationships between biomarker keywords (cluster 1) and physiological process keywords (cluster 2) only keywords labeled as “bioentity” and “process” were retained. The other keywords (resources, such as “cohort analysis” or “procedures” or similar) are not informative in this type of analysis and therefore were removed from the network, leaving 45 keywords as nodes, represented in Figure 6.

The contexts in which selected single bioentity keywords appear consist of the connected first neighbors of the biomarker node. The three most frequent keywords in the analysis were “inflammation” “metabolism” and “cytokines.” The “cytokines” keyword is connected to all nodes (Figures 5, 6). Other bioentity keywords form more specific connections. For example a “creatinine” keyword is connected to “inflammation” “skeletal muscle” “muscle strength” and “muscle mass” “oxidative stress” “metabolism”; the “heat shock protein 72” keyword is connected to the “muscle atrophy” “muscle growth” “inflammation” “metabolism” and “aged 80 and over.” Interestingly the keywords “gastrointestinal microbiome” and “intestine flora” are connected to “muscle mass” “metabolism” “physical performance” “inflammation” “aging” and only to one bioentity – “cytokines.” Since the number of keywords is not big all connections were explored. Connected biomarkers are summarized in Table 2.

**Table 2:** Connected biomarkers keywords (in two clusters) and contexts of the molecular mechanisms of sarcopenia and physical frailty.

CLUSTER 1: biomarkers	Total link strength	CLUSTER 2: physiological processes and another related biomarkers* from CLUSTER 1	References SCOPUS
*Cytokines*	470	Mitochondrial dysfunction, skeletal muscle, muscle mass, muscle strength, muscle atrophy, inflammation, oxidative stress, metabolism, insulin sensitivity, disability, proteomics, protein expression, protein degradation, physical performance, pathophysiology, aged 80 and over; *interleukin 6, myostatin* *	(11, 24, 28–40)
*Interleukin 6*	356	Inflammation, oxidative stress, insulin sensitivity, metabolism, skeletal muscle, muscle atrophy, muscle strength, grip strength, muscle mass, body composition, proteomics, protein expression, protein degradation, pathophysiology, physical performance, aging; myokines, *cytokines, C reactive protein, creatinine, tumor necrosis factor* *	(11, 24, 26, 27, 30, 33, 37, 40–55)
*C reactive protein*	259	Metabolism, inflammation, skeletal muscle, proteomics, protein expression, protein degradation, physical performance, oxidative stress, muscle strength, muscle atrophy; *tumor necrosis factor, interleukin 6, cytokines* *	(24, 33, 37, 40, 56)
*Creatinine*	249	Skeletal muscle, muscle mass, muscle strength, metabolism, inflammation, oxidative stress, pathophysiology, aged 80 and over; *cytokines* *	(11, 30, 32, 36, 57–59)
*Tumor necrosis factor*	203	Inflammation, muscle atrophy, physical performance, muscle strength, very elderly; *C reactive protein, cystatin C* *	(24, 30, 32, 33, 37, 43, 49, 50, 60)
*Amino acids*	185	Metabolism, inflammation, muscle mass, physical performance, very elderly, aged 80 and over; *cytokines, citrulline, aspartic acid, asparagine* *	(11, 24, 34, 49, 61–65)
*Citrulline*	150	Metabolism, inflammation, muscle strength, muscle mass, physical performance, metabolomics, very elderly; *cytokines, myeloperoxidase, heat shock protein 72* *	(11, 29, 43, 50, 54, 62–65)
*Myeloperoxidase*	148	Inflammation, physical performance, aged 80 and over; *cytokines, interleukin 17, heat shock protein 72, macrophage inflammatory protein-1 alpha, platelet derived growth factor BB, citrulline* *	(11, 24, 26, 27, 29, 50, 56)
*Heat shock protein 72*	139	Muscle atrophy, muscle growth, inflammation, metabolism, pathophysiology, aged 80 and over; *myeloperoxidase* *	(11, 24, 26, 27, 29, 43, 62)
*Macrophage Inflammatory Protein-1 beta*	134	Inflammation, very elderly, aged 80 and over; *cytokines, platelet derived growth factor BB* *	(29, 33, 34, 56)
*Interleukin 17*	114	Physical performance, inflammation, aging; *cytokines, interleukin 8, myeloperoxidase* *	(11, 26, 27, 35, 56)
*Interleukin 7*	112	Mitochondrial dysfunction, metabolomics, inflammation, extracellular vesicles, aging; *cytokines* *	(11, 26, 27, 31, 35)
*Hemoglobin*	99	Muscle, inflammation; *cytokines, macrophage inflammatory protein-1 alpha* *	(24, 32, 36, 49, 50)
*Platelet derived growth factor BB*	85	Inflammation, aged; *cytokines, myeloperoxidase, macrophage inflammatory protein-1 alpha* *	(29, 50, 56)

*Biomarkers (i.e., biomolecules, proteins) from cluster 1 that interact with each other are marked in Italics.

All biomolecules (in cluster 1) interact with each other and are related to the keywords – “biomarkers” and “biological markers.” In addition, all biomolecules are related to “metabolism,” “inflammation,” “metabolomics” and many of them are to “muscle” (such as “muscle mass,” “muscle strength”). Overall the connections between the retrieved keywords and their occurrences reveal one overarching context which is “inflammation” and “cytokines.”

## 4 Discussion

Physical frailty and sarcopenia are closely connected clinical conditions that correlate with the aging process in musculoskeletal system and indicate the increased risk of vulnerability and negative health related outcomes. Though the loss of skeletal muscle strength and mass seem to be progressively unavoidable, it can be in part avoided or averted by further understanding of the biological processes and pathogenesis of PFS. Specific mechanisms and biomarkers underlying these conditions are not completely clear and are currently under investigation. Our bibliometric analysis outlined the main directions and aspects of the most cited studies on molecular mechanisms and biomarkers of PFS, alternative to previous studies that informed on PFS in general or in a certain aspect of these two conditions. Understanding the attributes, hotspots, and trends of reported studies is fundamental due to the fact that they refer to the underlying developments in the field of PFS. Our novel contribution to the literature analysis is that we present biomarker keywords (for PFS) in relation to other keywords potentially designating processes and mechanisms, and reveal the biomarker identities and current contexts in which these biomarker identities are discussed.

Our study showed that the research of biomarkers for PFS went up progressively in the last 8 years from 2015 to 2023, and an increase in the number of scientists focused their attention on this field. In general, the number of academic paper relevant to the molecular mechanism and biomarkers for PFS has steadily expanded through the years and reached a peak in 2017–2020. High citation of quite recent papers shows very active research interest in this topic. The results of the article citation analysis revealed that all of the high-cited sources are the top journals in the field (such as “Aging Clinical and Experimental Research,” “Experimental Gerontology” etc.), which may offer more competent research sources for analyzing the particular pathways of PFS. Moreover, we found that Italy and the United States of America are the leading countries in terms of contributions to the development of this topic. The SPRINTT Consortium published more than 30 articles on PFS in older people.

Thus far, the recognition of PFS depends on clinical and functional parameters (11). For instance, the physical phenotype of frailty (according to Fried’s criteria), for example lower handgrip strength, decreased gait speed, and lower physical activity show a vast overlay with the features of sarcopenia (1–9). However, our study found that biological indicators relevant to different fields (e.g., muscle atrophy, inflammation, cytokines, metabolism) have been shown as possible biomarkers for these conditions. Most prevalent themes focus on biomarkers, inflammation, metabolism, muscle function, physical performance, the aging process, cognitive function, long-term care, and nutrition.

The scientific publications showed that PFS are very complex syndromes where many physiological and biochemical changes happen to the older person that influence all levels of the organism (from cell to organ systems) and cause a large variety of changed molecular processes (5–9, 11, 26, 27, 35, 66). Both frailty and sarcopenia are characterized by a subacute pro-inflammatory condition that affects metabolism and destroys the functioning of skeletal muscle (11, 39, 47). Furthermore, other contributors to muscle dysfunction are reduced neuronal stimulation, mitochondrial dysfunction, oxidative stress, and inflammation (11, 39, 44, 47, 52, 53, 55). A variety of experimental and clinical studies had described continuous denervation and partial reinnervation of skeletal muscles even though the underlying mechanisms of age related motoneuron loss include a number of not fully understood factors. In addition some hallmarks of aging such as mitochondrial dysfunction, loss of proteostasis and inflammatory disturbances are the key mechanisms currently understood to be involved in PFS development (11, 26, 27, 44, 47, 48, 51, 52).

Most of the studies showed that the molecular and cellular mechanisms of PFS involve an crosstalk between multiple signaling pathways. The beginning of muscle loss is created by an inadequacy between muscle protein synthesis [the key signaling pathway is PtdIns-3-OH kinase-(PI3K)-AKt, insulin-like growth factor-1- (IGF-1)] and proteolysis (including ubiquitin-proteasome pathway), and interconnected with autophagy (the autophagy-lysosomal pathway) (53, 55). Recent studies showed that cytokines or myokines (synthesized and released by myocytes during muscle contractions) dysfunction can cause and aggravate PFS (44, 47, 48, 51, 52). Myokines carry out autocrine, paracrine and endocrine signaling acts (triggering specific molecular pathways in different tissue and organs) and control a few processes correlated with muscle wasting, dynapenia (loss of muscle strength), and low physical functioning (24, 26, 27). For example, interleukin-6 (IL-6) can be produced by most of the cell type and various tissues under right circumstances, and many researches have studied IL-6 as a pro-inflammatory cytokine with major relationship with lower muscle mass, muscle weakness, and sarcopenia (44, 52). Remarkably, myokine synthesis and expression by the skeletal muscle are prompted under both anabolic and catabolic pathways, with systemic and local consequences (24, 26, 27, 47). For example, myokines (such as IGF-1, myostatin, irisin, decorin) can reduce resistance to insulin and correlate with muscle wasting and dynapenia (24, 26, 27, 47, 67). Furthermore, the sarcopenic muscle exhibits upturn in myostatin signaling and therefore starts a process of advanced muscle loss (26, 48). Some studies revealed a significant relationship between myokine and phenotype of sarcopenia and/or physical frailty, such as myostatin and IL-15 associated with muscle weakness and/or wasting, IGF-1 with muscle weakness, slowness, disability (24, 26, 27, 47). Taken together, myokines (especially cytokines) show the promise to be used as biomarkers of frailty and this occurrence seems to apply to sarcopenia as well (24). However, only several myokines have been researched in relation to PFS.

Recent research suggests that immunosenescence (alteration of immune functions due to aging) and inflammaging are two key age associated processes essentially causing an accelerated aging and multidimensional PFS (47, 50, 51, 54). Immunosenescence affects both innate and adaptive immune response (46). Part of immunosesenescence is inflammaging, a term meaning that long term low grade inflammatory activity leads to multiple tissue damage (42). Inflammaging is associated with higher concentrations of inflammatory markers (such as cytokines), for instance IL-6, IL-1, and tumor necrosis factor (TNF) (41, 43, 44, 51, 52, 54). Increased levels of IL-6 were also found in older patients with sarcopenia (depending on the severity of sarcopenia) (51, 52). A systematic review and meta-analysis conducted by Tuttle et al. (38) revealed that increased levels of IL-6, TNFα, and C reactive protein (CRP) were related to lower handgrip strength and high CRP were associated with decrease in muscle mass. Similarly, increased IL-6, TNFα, and CRP levels were correlated with pre-frailty and frailty (45, 54). Taken together, the association between the immunity and aging is complicated, however, studies show that inflammaging is associated with a higher risk of PFS.

According to existing research, frailty is the key component of an increased vulnerability to chronic diseases in older adults (1, 4, 5, 31, 59). Many of frailty biomarkers have been identified in predicting vascular aging and cardiovascular diseases on cellular or even molecular levels. Pisano et al. stated that the proper nutrition and moderate-intensity resistance training are crucial for better preparing patients undergoing cardiovascular procedures (59). The studies about protein intake and chronic age-related conditions showed that disorders in protein–amino acid metabolism may substantially affect the pathophysiology of sarcopenia (64, 65). Additionally, the proteins participating in the calcium signaling pathways (and calcium-binding proteins) are important biomarkers of cellular, tissue, and systemic dysfunction in older adults (68–71). Calvani and colleagues investigated specific multi-marker datasets through a novel research strategy based on SO-CovSel (Sequential and Orthogonalized Covariance Selection) for PFS biomarkers selection (including mediators of inflammation, metabolism, and mitochondrial dysfunction) (28, 29, 60–62). They identified the number of discriminants PFS biomarkers: aspartic acid, asparagine, citrulline, α-aminobutyric acid, platelet derived growth factor BB (PDGF-BB), myeloperoxidase (MPO), and heat shock protein 72 (Hsp72), but these results need to be validated in longitudinal studies (29).

Calvani and colleagues reported that a lower serum level of Hsp72, MPO, Macrophage Inflammatory Protein -1β (MIP-1β) and PDGF-BB are present in individuals with PFS (29). The reduced circulating levels of chemokine MIP-1β (regulate myoblast functioning) may indicate a disturbance in macrophage polarization (an important process for muscle regeneration) (29). Micronutrient deficiency (such as iron) is common in PFS older adults and can cause secondary decrease in MPO levels. Satellite cells and myoblast proliferation is stimulated by PDGF-BB which performs an important part in the regeneration of skeletal muscle mediated by platelets. Therefore, lower levels of PDGF-BB may indicate a defective regenerative potency of skeletal muscle in PFS pathogenesis. Moreover, hormetic signals are needed to protect muscle function and mass but disruption in this process can be rated to the lower levels of Hsp72. Plasma concentration levels of Hsp72 were associated with low muscle strength and mass in older adults and for this reason Hsp72 has been suggested as a potential biomarker for Sarcopenia (29). Consequently, the reduced concentration of Hsp72, PDGF-BB, MPO and MIP-1β may be thought of as a particular immunosenescence markers for PFS (29). The occurrence of citrulline, aspartic acid and asparagine among the candidate biomarkers of PFS may represent the perturbations in a several tissue-specific and inter-organ processes regulating cellular anabolic pathways, energy metabolism, and micronutrient balance (29).

The data suggest that sarcopenia and frailty have a different specific metabolic pathway. Sarcopenia can be described as aging of the muscle with a reduction of metabolites for mitochondria, kidney, muscle, and methylation, compared to the drop in metabolites for antioxidation in frailty (31, 60–63). Interestingly that during our bibliometric analysis, an association of the “proteomics,” “metabolomics” and “metabolism” keywords with the PFS was obtained. Actually, metabolomics is a novel systems approach for researching metabolic profiles (small molecules and their interactions within a biological system), which gives information on illness pathogenesis and gene function at different metabolic pathways (53, 72). Specific environmental factors, physiological conditions, and gene expression can cause alteration in occurring homeostatic states, which are mirrored in the metabolic pathways of the various diseases, as well as PFS. That is why metabolomics is a useful approach for exploring molecular mechanisms and changes in metabolic processes (e.g., in protein, energy balance, oxidative stress, mitochondrial fatty acid oxidation, lipid metabolisms, and muscle function), and then linking these metabolomic data to phenotypic profile. This allows for the ability to present the entanglement of the aging process and conditions such as PFS (72). This investigation plan supports the complicated course of recognizing biomarkers characteristic of the individual reaction to particular physiological or environmental factors (such as nutritional or physical interventions) (72–74). Since intervention programs (such as physical exercise, cognitive training, supplementation, and nutrition) are more likely to be most effective in the early stages of PFS, the uncovering of high sensitivity and specificity biomarker(s) for the early identification of these conditions is extremely important.

Recently, the importance of gut microbiota in prompting concrete metabolic conditions in older adults has been reported by the widening scientific evidence. For this reason, it is important to understand underlying molecular processes of the effect of gut microbiota on PFS (27, 29, 34).

It should also be noted that there has been a rise in the number of articles on the genetics of PFS, especially in the last 7 years from 2017 to 2023 (53, 73–80). Such studies contribute to a better comprehension of the molecular mechanisms fundamental to the progression of PFS, and in the future, may advice to delay or even avoid such conditions (75). Recently, 78 genomic predictors of severe sarcopenia (i.e., associated with all three criterion — low muscle strength, low muscle mass, and slow gait speed — with persistent directions of effect) have been discovered (81). Interestingly, according to the UK Biobank database and two recent genome-wide association studies on frailty (75, 80), 17 of those 78 single nucleotide polymorphisms (SNPs) are also related to frailty index or frailty score, and another 27 SNPs are linked with other symptoms of frailty, such as tiredness, low physical activity, falls in the last year, and neuroticism (depressive symptoms). These data indicate that PFS share many common risk alleles (75, 76, 80).

Definitely, the identification of biomarkers, through system biology perspective (using genomics, proteomics, and other omics), aiding in new strategies for diagnosis and treatment of age-related PFS are vital for the understanding of the essential biological processes and molecular mechanisms of PFS (especially reduced muscle mass). The general goal is to avert or slow down the decrease of the main physical functions necessary to live a full, independent life (48, 53, 55).

There are several limitations of the present study. The bibliometric analysis focuses on the relationship between publications to give a landscape of work done in a specific field. However, the bibliometric analysis does not evaluate the quality of the research. Also, only the terms “sarcopenia” and” physical frailty” were included in this analysis which might exclude research related to prefrailty or research focusing on low muscle mass and strength. Moreover, although a significant number of biomarkers (biomolecules) were found, we could not find all potential mediators associated with PFS.

This study has a few strengths including the use of a new bibliometric analysis of all available studies to evaluate the relationship and the ability to harmonize the literature data into consistent findings to enhance pooling of knowledge on the pathophysiology and molecular mechanisms of PFS.

## 5 Conclusion

The present study summarized the global research trends of biomarkers and molecular mechanisms for PFS over the past 2 decades (1997–2023) using bibliometric analysis. Given the relevance of increased vulnerability to negative health-related outcomes of sarcopenia and frailty in clinical trials and research, biomarkers and molecular mechanism of these conditions have been actively sought after. The exploration of biomarkers of PFS has been a growing and engaged area of research. Many published articles analyzed the background of the PFS, and biomarkers are pertinent goals in clinical decisions and randomized clinical studies. Biomarkers serve to realize the underlying pathophysiology of PFS. In the recent times, extending research interest in this field of PFS has been focused on the inflammatory process and probably will be designated to myokines (cytokines and other small proteins) that are synthetized and released by skeletal muscles in response to physical activity. Although various candidate biomarkers have been revealed, none of them have yet been included in the diagnostic or follow-up process of PFS. Considering that changes in biomarkers may come before the clinical presentation of these conditions, it’s possibly allowing timely corrective interventions. In general, our study may elucidate new frontiers in the diagnosis or prevention of these age-related conditions. Conceivably, in the future, combining multiple stratification of genetic biomarkers will be demanded to provide a more precise prediction of PFS and to evaluate the outcomes of PFS interventions.

## Data availability statement

The original contributions presented in the study are included in the article/supplementary material, further inquiries can be directed to the corresponding authors.

## Ethics statement

Ethical approval was not required for the study involving humans in accordance with the local legislation and institutional requirements. Written informed consent to participate in this study was not required from the participants or the participants’ legal guardians/next of kin in accordance with the national legislation and the institutional requirements.

## Author contributions

VG: Conceptualization, Data curation, Methodology, Writing – original draft, Writing – review & editing. EP: Data curation, Investigation, Methodology, Software, Writing – review & editing. JK: Conceptualization, Investigation, Methodology, Writing – original draft, Writing – review & editing. AM: Conceptualization, Formal analysis, Investigation, Writing – original draft. RD: Conceptualization, Formal analysis, Writing – original draft. IJ: Formal analysis, Writing – original draft. AL: Data curation, Methodology, Software, Writing – original draft. IA: Conceptualization, Supervision, Writing – original draft. VA: Project administration, Writing – review & editing.
